# COMMUNITY PARTNERSHIPS: A MODEL FOR LIFELONG DISABILITIES AND DEMENTIA CARE

**DOI:** 10.1093/geroni/igz038.2756

**Published:** 2019-11-08

**Authors:** Faith Helm, Catherine Taylor, Phillip G Clark

**Affiliations:** 1 Rhode Island Geriatric Education Center at the University of Rhode Island, Kingston, Rhode Island, United States; 2 Rhode Island Geriatric Education Center, Kingston, Rhode Island, United States

## Abstract

In 2015 the Rhode Island Geriatric Education Center (RIGEC) was one of 44 organizations funded by HRSA to implement the Geriatrics Workforce Enhancement Program (GWEP). A primary objective was to develop and deliver Alzheimer’s Disease and Related Dementias (ADRD) education to patients, families, caregivers, and health professionals, with a focus on special populations. Concurrently, Seven Hills Rhode Island, a nonprofit agency that serves people with disabilities, received a grant from the Administration for Community Living (ACL) to provide education and resources to health professionals and caregivers of people with I/DD. As goals for both projects aligned closely, they worked together, fostering a strong partnership, amplifying the opportunity to offer high-quality educational programs and reach target audiences. Lessons learned from this networked approach are critical to informing sustained improvements to the I/DD and health care systems in subsequent GWEP projects.

